# Distinguishing Gender Identity From Biological Sex in Dermatologic Health Care: Methods, Harms, and Paths Forward

**DOI:** 10.2196/47118

**Published:** 2023-07-18

**Authors:** Noah N Nigro, Neal Chandnani, Athena Doshi, Alexa Fritsch, Nathaniel A Marroquin, Morgan Zueger, Torunn E Sivesind, Robert Dellavalle, Cory Dunnick

**Affiliations:** 1 Department of Dermatology University of Colorado School of Medicine Aurora, CO United States; 2 Rollins School of Public Health Emory University Atlanta, GA United States; 3 Rocky Vista University College of Osteopathic Medicine Parker, CO United States; 4 Dermatology Service Rocky Mountain Regional Medical Center Eastern Colorado Health Care System Aurora, CO United States; 5 Dermatology Service US Department of Veterans Affairs Aurora, CO United States; 6 Department of Epidemiology Colorado School of Public Health Aurora, CO United States

**Keywords:** gender identity, biological sex, gender differences in health, dermatologic health, literature representation, sex-gender bias, gender assessments, skincare, communication strategies, health care questionnaires, healthcare questionnaires, dermatology

## Abstract

Accurate assessment of gender identity and biological sex in dermatology research is crucial since their conflation or poor demarcation undermines patient respect and study accuracy. Clearer guidance is needed for health care researchers, particularly in light of the notable disparities in skin disease rates, skincare practices, literature representation, and the persistent underrepresentation of transgender and nonbinary patients.

## Introduction

Although gender identity is a social determinant of health, its assessment in health care research is inadequate. We highlight the intricacy of gender and biological sex in dermatology research, revealing the need for more robust protocols for their assessment. We begin by evaluating the current protocols used to make such assessments, demonstrating lack of consensus. Next, we evaluate the relationships between biological sex and gender identity and how these impact skin health. We then examine the inadequate representation of gender minorities—including those who identify as transgender or gender nonbinary—in academic literature and how this disparity compromises the applicability of evidence-based medicine to all. Finally, we consider the importance of physician communication about gender identity.

## Methods

The articles analyzed in this study emphasize the importance of transparency when delineating differences between biological sex and gender identity. In addition, researchers should be coached on techniques to extinguish investigators’ biases. The models and tools for discerning gender identity and biological sex are shown in Textbox 1.

Note that the results obtained from the above methods were analyzed in conjunction with other questionnaires to assess correlations between the factors affecting overall population health [1,2,4]. Gender identity assessment tools must be regularly updated to accurately reflect the specific generational, cultural, and institutional contexts of the time.

**Tools for discerning gender identity and biological sex.**

**Questionnaires**

- Stanford Gender-Related Variables for Health Research (GVHR) [1]
- Bem Sex-Role Inventory (BSRI) [1,2]
- BSRI: short form [1,2]
- GENESIS-PRAXY (Gender and Sex Determinants of Cardiovascular Disease: From Bench to Beyond-Premature Acute Coronary Syndrome) [1]

**Guidelines and recommendations**

- Sex and Gender Equity in Research (SAGER) guidelines [3]
- Sex-gender variable: methodological recommendations for increasing scientific value of clinical studies [4]

## Results

### Gender Differences in Dermatologic Health

Cisgender (gender identity corresponds to sex assigned at birth) males and females exhibit different rates of various skin diseases [5]. Increased awareness of gender differences is central to constructing mechanisms for prevention, diagnosis, and therapy [5]. Those assigned male at birth tend to have higher sebum content, thicker skin, and deeper facial wrinkles than those assigned female [6]. However, research regarding gender and dermatologic care is limited. Most articles discuss gender in terms of cisgendered participants. This research is likely based on the conflation of biological sex and gender identity in electronic health records [7]. Systemic change within electronic health records is a critical step for gathering gender-precise data.

Gender-affirming therapies may induce drastic skin changes. Hormone therapies increase the risk of acne vulgaris, androgenetic alopecia, excessive sebum production, melasma, and hirsutism [8]. Dermatologists must be conscious of gender-affirming treatments in order to help minimize associated risks.

### Gender Minorities in Literature Representation

Researchers believe numerous factors contribute to skin differences between gender identities [8]. Transmale and transfemale populations (gender identity does not correspond with sex assigned at birth) face underdiagnosed conditions resulting from underresearched gender-affirming therapies [8]. This representation disparity is a growing area of investigation for its contribution to gender inequality.

Gender minority populations also face health care barriers from noninclusive treatment eligibility models. For instance, despite the increased prevalence of acne vulgaris in transgender populations, iPledge medication guidelines for isotretinoin use sex assigned at birth and require contraception to remain eligible [8].

Although increasing female participation in clinical trials is widely supported, data on gender minority participation is nonexistent. Additionally, the risks of some conditions fluctuate depending on the stage of gender-affirming therapies. Although existing data sets may not use inclusive models of gender identity assessment, there is value in their implementation given the prospect of large-scale observational studies and meta-analyses in which these variables could be better assessed. As gender minority populations grow, these inclusive models will help physicians support all patients while ensuring that salient trends and health impacts are captured by research analyses.

### Physician Communication

**Figure 1:**
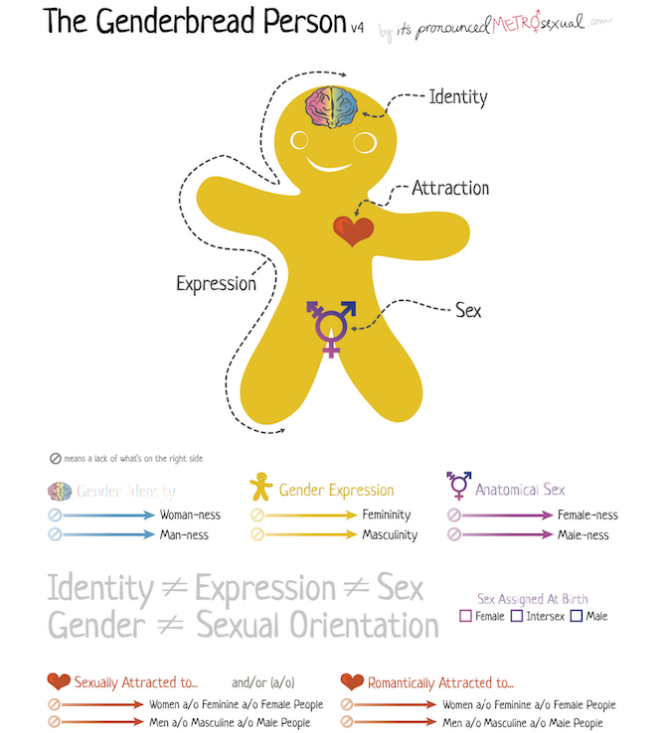
A “Genderbread Person” outlining differences in gender identity, expression, and sex [10].

About 28% of transgender individuals delay seeking preventative care because of mistreatment by health care workers; thus, inclusive language is important to build trust in these communities [9]. Medical professionals should receive training in gender-inclusive terminology to foster a welcoming environment where patients feel comfortable sharing information relevant to their health [7]. Physicians should avoid assumptions and ask patients about their preferred terminology [7]. Figure 1 displays a “Genderbread Person” and provides guidance on the terminology used in this paper.

Misgendering patients can have profound effects on patients’ health and safety and can damage the patient-provider relationship [11]. As ideas about gender identity evolve, mistakes will inevitably happen; when mistakes occur, sincere and brief apologies should be made, with an assurance that they will not recur [11].

## Discussion

Robust methodologies exist to eliminate subjectivity and maximize data accuracy and utility. Current approaches to distinguishing gender identity and biological sex are inadequate and threaten the applicability of research findings to many patients. Conflating sex and gender neglects the unique dermatologic health impacts of these attributes and contributes to the underrepresentation of gender minority populations in medical literature. While more research is needed to address these issues, communication training for physicians and other health care providers could be improved. The language used must respect patients’ identities while maintaining objectivity in clinical research.
